# The combined antimicrobial effect of arginine and fluoride toothpaste

**DOI:** 10.1038/s41598-019-44612-6

**Published:** 2019-06-10

**Authors:** Mohammed Nadeem Ahmed Bijle, Manikandan Ekambaram, Edward C. M. Lo, Cynthia Kar Yung Yiu

**Affiliations:** 1 Paediatric Dentistry, Faculty of Dentistry, The University of Hong Kong, Hong Kong, SAR Hong Kong; 2 Faculty of Dentistry, University of Otago, Dunedin, New Zealand; 3 Dental Public Health, Faculty of Dentistry, The University of Hong Kong, Hong Kong, SAR Hong Kong

**Keywords:** Bacteria, Paediatric dentistry

## Abstract

The aim of the study was to investigate the antibacterial effect of arginine (Arg) in NaF toothpaste. 24-h mono-/3-species biofilm cultures of *S. mutans, S. sanguis and S. gordonii* inoculated sHA discs were subjected to treatment with toothpaste supernatants prepared as - [1]:2% Arg –NaF (0.147% F), [2]:4% Arg–NaF (0.144% F), [3]:8% Arg – NaF (0.138% F), [4]:NaF (0.15%) and [5]:deionized water. After 24-h incubation, the mono-species biofilms were subjected to viability assay using WST-8, SEM and confocal imaging (CLSM). The 3-species biofilm were quantified for bacterial composition by PCR analysis, SEM, CLSM, and RNA isolation with reverse-transcription PCR analysis. Increasing arginine concentrations in NaF toothpaste had no effect on microbial viability. The mono-/3-species biofilm imaging depicted that the 2% Arg-NaF and 4% Arg-NaF had a biofilm disrupting effect. The 3-species biofilm bacterial composition indicated that the 2% Arg-NaF group maintained an ecological homeostasis by inhibiting *S. mutans* growth and enriching the growth of *S. sanguis* and *S. gordonii*. The 2% Arg-NaF group significantly downregulated the expression of virulent *gtf*B gene and upregulated the expression of *sag*P with relative dominance of *arc*A. Incorporation of 2% arginine in NaF toothpaste might enrich the alkali-producing bacteria and provide enhanced counter mechanisms against cariogenic pathogen when compared to NaF toothpaste.

## Introduction

Considering the global burden of untreated dental caries, especially in the high risk population, the alarming need for caries prevention is evident^1^. The biofilm-mediated acidogenic/aciduric microbial shift is a proposed factor for the initiation of the caries process. The ecological shift reciprocally suppresses the oral commensals that prevails in the microflora in healthy conditions^2^. The commensals constitute *S. sanguis* and *S. gordonii* (alkali-producing bacteria) that neutralize pH by utilizing salivary/plaque arginine through the arginine deiminase system (ADS)^3,4^. Hence, caries preventive measures directed towards the enrichment of alkali-producing bacteria with concurrent suppression of cariogenic bacteria (e.g. *S. mutans*) is potentially beneficial^3–5^.

Fluoride is a known anti-caries agent; however, it has little sustained effect on cariogenic biofilms^5,6^. A limited antimicrobial effect of sodium fluoride (NaF) at ≥1200 ppm fluoride on *S. mutans* biofilms has been reported^7,8^. The effect of fluoride on bacterial metabolism is theorized by inducing cytoplasmic acidification and glycolytic enzymes inhibition^9^. However, a recent study advocate that the presence of biofilms influences the outcome of anti-caries agent^10^. Studies have also shown that the presence of biofilms negatively influences NaF anti-caries treatment outcomes^8,10^. Additionally, long-term use of fluorides has led to the evolution of fluoride-resistance *S. mutans*^11,12^. Therefore, augmenting the antibacterial effect of fluorides with biofilm modifiers may help to counter the concerns without reducing its remineralization potential^13^.

The use of arginine, a prebiotic amino acid, has been shown to affect the oral biofilms ecology^14–16^. Primarily, *S. gordonii* and *S. sanguis* metabolize arginine into ornithine, citrulline, ammonia, carbon dioxide and ATP through ADS. These oral bacteria which are associated with a biofilm compatible with health utilize the metabolic byproduct (ATP) for their survival; thereby promoting ecological homeostasis^3^. Furthermore, presence of these bacteria and their metabolites inhibits the growth of cariogenic bacteria due to a non-conducive alkalogenic environment^17^. Several studies have found a significant association between saliva/plaque ADS activity and dental caries which suggests that an increase in ADS activity leads to a decrease in dental caries^18–21^. In addition, external supplementation of arginine will increase ADS activity. Thus, upregulation of ADS activity by arginine as a biofilm modifier appears promising.

So far, studies have investigated the effect of arginine in low concentration (≤500 ppm F) NaF solution reporting its synergistic effect in maintaining a healthy oral microbial equilibrium^22,23^. No studies have analyzed the antimicrobial effect of arginine in sodium fluoride solutions ≥1000-ppm, concentrations commonly present in commercial toothpastes. Although the commercially available arginine-fluoride toothpaste contains 1.5% arginine bicarbonate in Na-MFP at a fluoride concentration of 1450 ppm^24–28^, the optimum concentration of arginine in NaF toothpaste is yet unexplored. Albeit the evidence contemplates the prebiotic effect of arginine to counter dental caries; a previous study examining the remineralization potential of arginine in 1100-ppm fluoride toothpaste concluded that 2% arginine in NaF toothpaste significantly increased the remineralization of artificial incipient enamel lesion compared to the control toothpaste containing NaF alone^29^. Given the prebiotic effect of arginine, the microbial effect of arginine-NaF blend on multi-species ecology is worth further investigation. Thus, this study aimed to evaluate the effect of different concentrations of arginine in NaF toothpaste on mono-species/3(multi)-species biofilm, simulating environment in high caries-risk patients. The null hypothesis in this study was that incorporating arginine in NaF toothpaste does not have an additional antibacterial effect as compared to NaF alone.

## Results

### Test agent characterization

**Figure 1:**
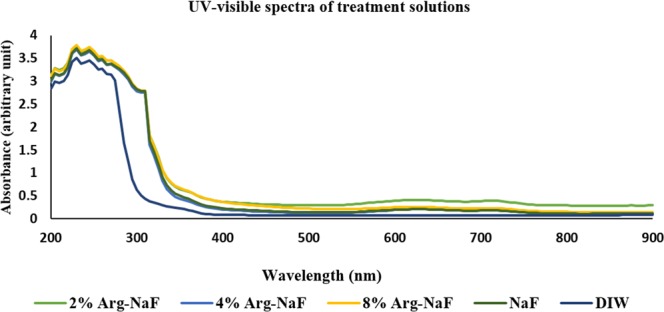
UV-visible spectra of test solutions.

Figure 1 shows representative mean UV-visible absorption spectra of different test agents characterized at different time-points in the entire experimental process. The characteristic peak for all the test agents fall within the UV spectra (200–400 nm). The presence of peaks in the UV spectra from all test agents suggested that the color change observed during the preparation of dentifrice slurries from control was non-contributory, since none of the characteristic peaks were measured in the visible spectra. Therefore, the test solution characterization advocated that incorporating arginine in NaF toothpaste might not cause any aesthetic-based deleterious effect to hard tissues.

### Mono-species biofilm microbial cell-viability

**Figure 2:**
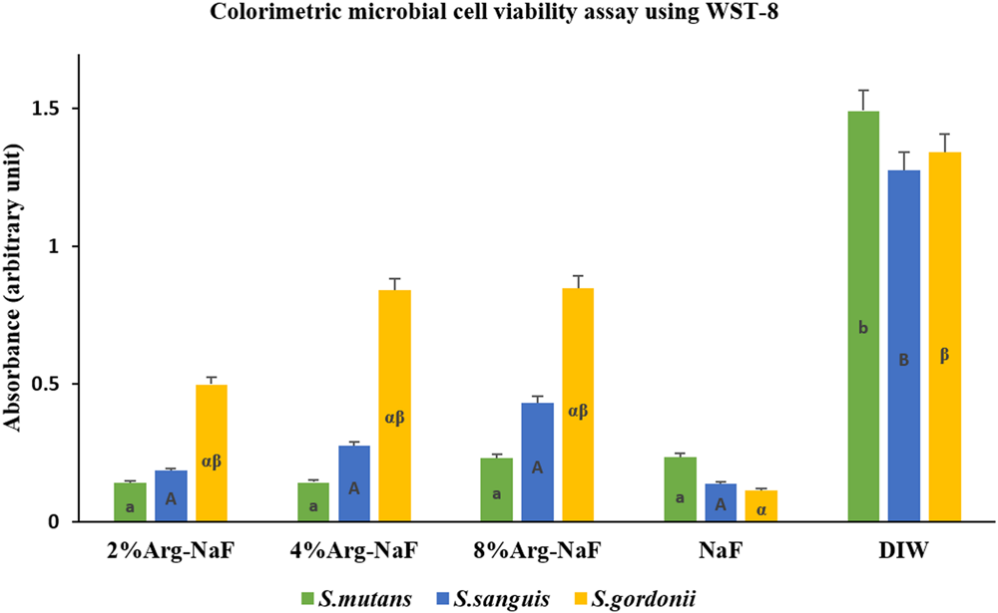
Microbial cell viability using WST-8 assay for mono-species - *S.mutans, S.sanguis, S.gordonii* biofilm subjected to different treatments (2%, 4%, 8% Arg-NaF, NaF, and DIW). The lowercase letters (a,b)/uppercase letters (A,B)/Greek alphabets (αβ) represent differences between different test groups as analysed by Kruskal-Wallis 1-way ANOVA followed by Dunn’s post-hoc test.

Microbial cell-viability of mono-species biofilm treated with different test agents is presented in Fig. 2. Kruskal-Wallis one-way ANOVA identified a statistically significant difference among the different test and control groups in individual mono-species biofilm (p < 0.001). All the dentifrice slurries had a significantly greater inhibitory effect on *S. mutans* and *S. sanguis* when compared to deionized water (DIW) (p < 0.05), with no significant difference among the different Arg-NaF dentifrice slurries and NaF (p > 0.05). Conversely, NaF has a significantly higher inhibitory effect on *S. gordonii* than DIW (p < 0.05). No significant difference in *S. gordonii* viability was observed among the different Arg-NaF dentifrice slurries and NaF (p > 0.05). Increasing the concentrations of arginine in NaF toothpaste has no effect on the viability of *S. mutans*, *S. sanguis* and *S. gordonii* in mono-species biofilm.

### Mono-/3-species biofilm imaging (SEM & CLSM)

**Figure 3:**
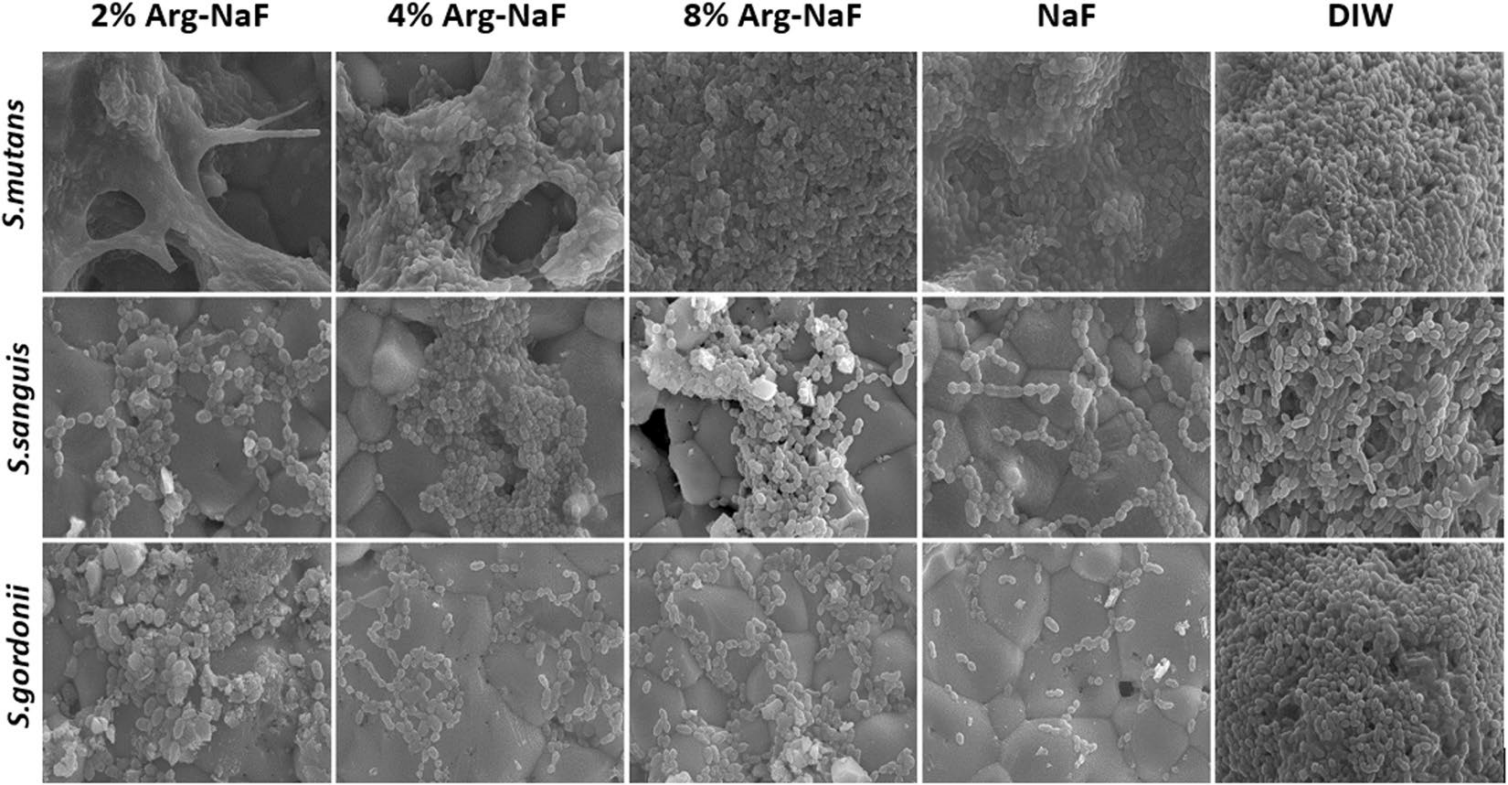
Scanning Electron Microscopy (6000x) of *S.mutans, S.sanguis, S.gordonii* mono-species biofilm subjected to treatments by test agents (2%, 4%, 8% Arg-NaF, NaF, and DIW).

**Figure 4:**
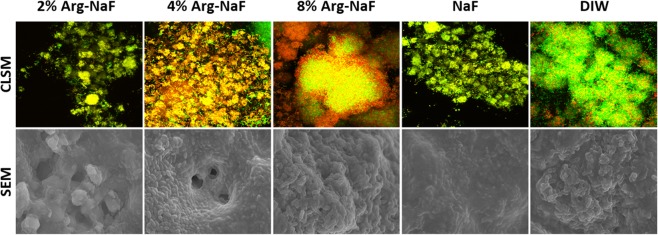
3-species (*S.mutans, S.sanguis, S.gordonii*) biofilm imaging (Confocal Laser Scanning Microscopy at 100x, Scanning Electron Microscopy at 6000x) subjected to different treatments (2%, 4%, 8% Arg-NaF, NaF, and DIW).

The SEM analysis showed that both 2% Arg-NaF and 4% Arg-NaF had a biofilm disrupting effect on *S. mutans* mono-species and 3-species biofilms (Figs 3 and 4). The biofilm surface topography for *S. mutans* mono-species and 3-species appeared similar for biofilms treated with 8% Arg-NaF, NaF, and DIW, revealing that these test solutions had minimal effect on cariogenic biofilms (Figs 3 and 4). The SEM images at 6000-x depicted that NaF inhibited the formation of *S. sanguis* and *S. gordonii* biofilms; whereas arginine-incorporated NaF upregulated the formation of *S. sanguis* and *S. gordonii* biofilms (Fig. 3).

**Figure 5:**
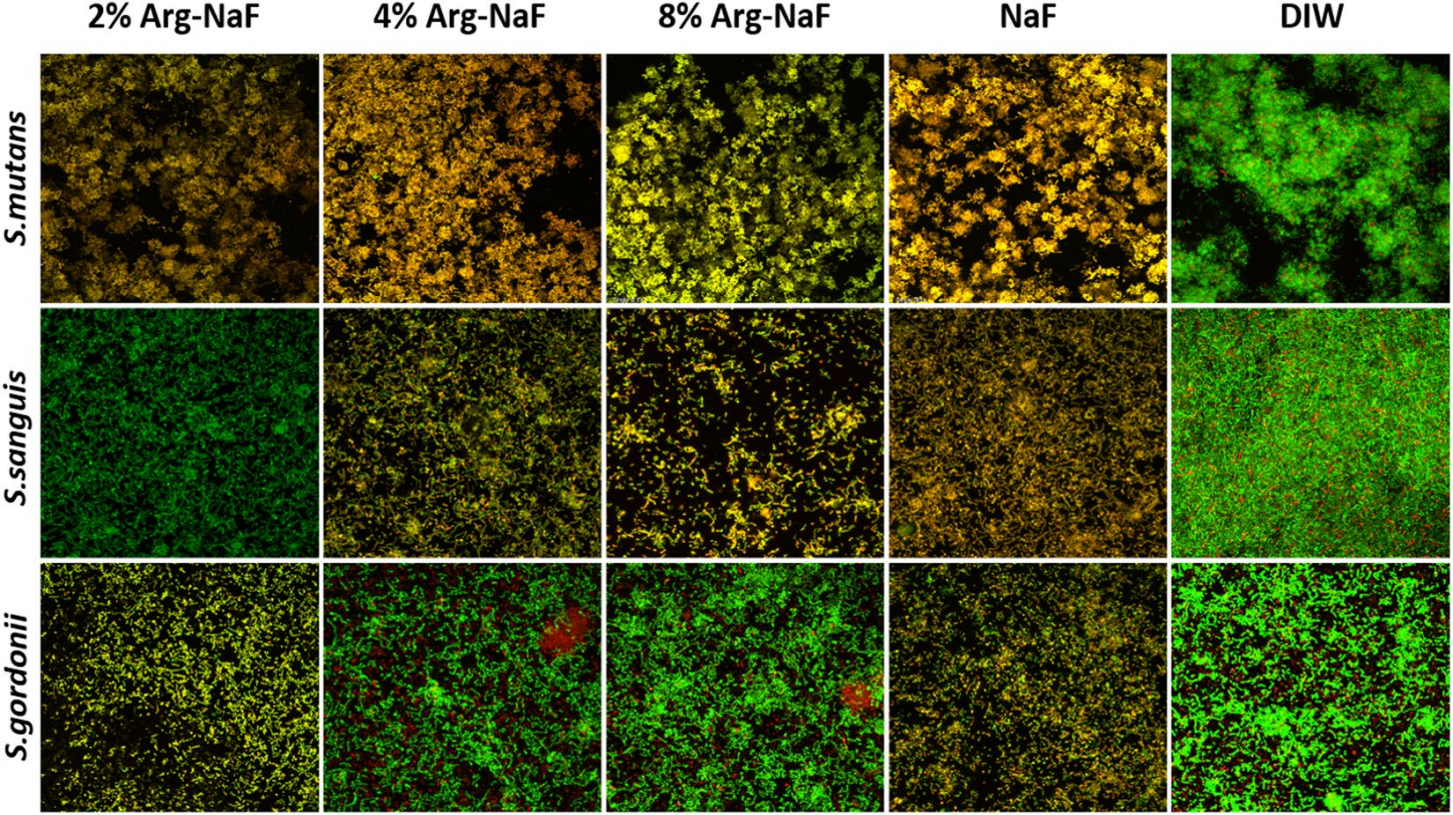
Confocal laser scanning microscopy (100x) of mono-species *S.mutans, S.sanguis, S.gordonii* biofilm subjected to different treatments (2%, 4%, 8% Arg-NaF, NaF, and DIW).

**Figure 6:**
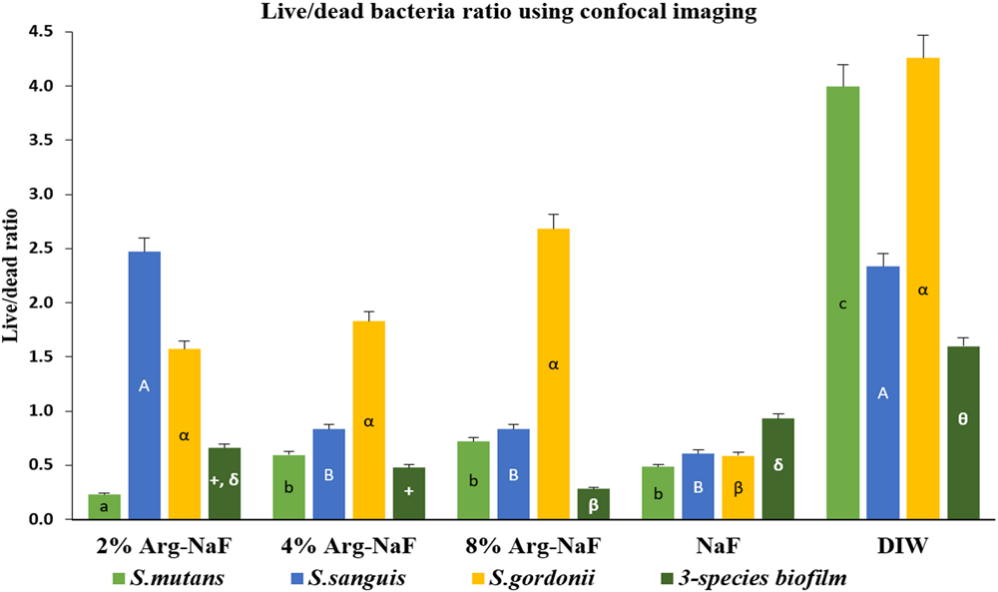
Live/dead bacteria ratio analyzed using confocal imaging (100x) for mono-/3-species biofilm with *S.mutans, S.sanguis, S.gordonii*. The difference between test agents (2%, 4%, 8% Arg-NaF, NaF, and DIW) treated biofilms for data analyzed with 1-way ANOVA followed by SNK test is represented by lowercase letters (a,b,c), uppercase letters (A,B), Greek alphabets i.e. α,β/Greek alphabets with signs i.e. +, δ, θ.

Figures 4 and 5 present confocal imaging (100-x magnification) of treated 3-species and mono-species biofilms, respectively. The live/dead bacteria ratio determined using the confocal images is shown in Fig. 6. One-way ANOVA revealed statistically significant difference in the log-transformed live/dead bacterial vitality ratio of the mono- and 3-species biofilms treated with different test agents (p < 0.001). For *S. mutans* biofilm, the live/dead bacteria ratio was significantly lower in the 2% Arg-NaF group than the other groups (p < 0.05). The *S. sanguis* biofilms treated with 2% Arg-NaF and DIW presented with significantly higher live/dead ratio when compared to 4% Arg-NaF, 8% Arg-NaF and NaF (p < 0.05). Conversely, the live/dead ratio for *S. gordonii* biofilms treated with Arg-NaF and DIW were significantly higher when compared to NaF (alone).

Overall, the mono- and 3-species biofilm imaging indicated that 2% Arg-NaF had a biofilm disrupting effect by inhibiting the growth of cariogenic *S. mutans* and it subsequently enriched the growth of *S. sanguis* and *S. gordonii*. Although both 4% Arg-NaF and 8% Arg-NaF enriched the growth of *S. sanguis* and *S. gordonii*, their inhibitory effect on *S. mutans* was lowered. Moreover, in the 3-species biofilm, the live/dead bacterial vitality ratio of 2% Arg-NaF is similar to that of NaF, suggesting comparable vitality of biofilm enriched bacteria as that of control.

### 3-species biofilm bacterial composition

**Figure 7:**
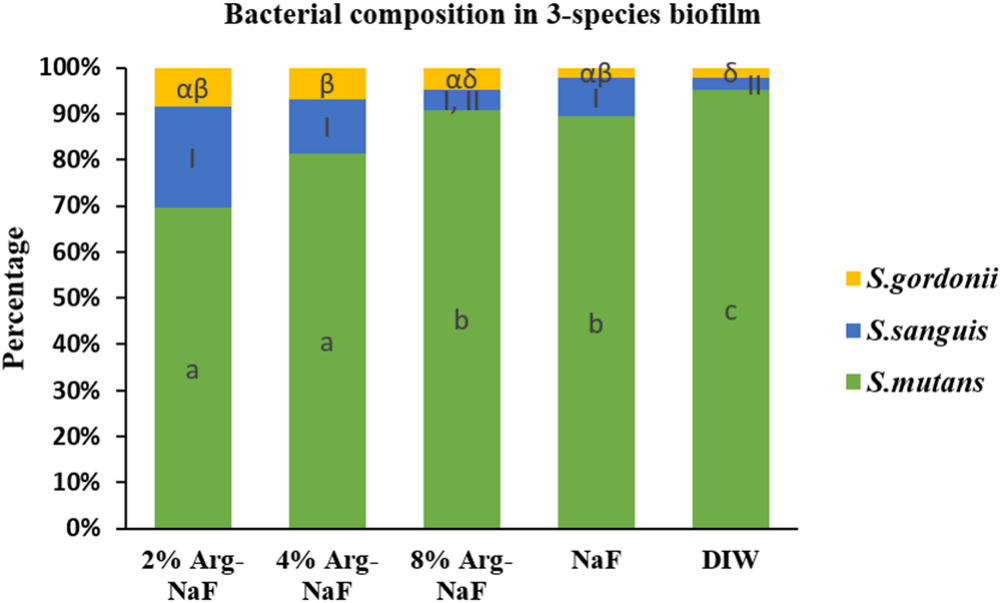
Effect on total bacterial composition in 3-species biofilm (*S.mutans, S.sanguis, S.gordonii*) treated with different test agents (2%, 4%, 8% Arg-NaF, NaF, and DIW) represented as proportions per treatment based on mean from 3 repeated time-points. The lowercase letters (a–c), numerals (I,II), Greek alphabets (α,β,δ) represent differences between different test groups as analyzed by 2-way ANOVA with Bonferroni’s correction.

The total bacterial composition in 3-species biofilm treated with different test agents determined by DNA extraction and real-time PCR analysis is shown in Fig. 7. The values obtained through PCR analysis were converted to CFU/ml. Figure 7 presents the mean proportional total bacterial composition of biofilms treated with different test agents at disparate time-points. The data was log transformed to satisfy the assumption of normality. Two-way ANOVA identified significant differences in the different groups (p < 0.01), bacteria (p < 0.001) and their interaction (p < 0.001). A statistically significant difference in the *S. mutans* composition was observed across the different test and control groups (p < 0.05). The 2% Arg-NaF and 4% Arg-NaF showed significantly higher inhibition of *S. mutans* growth when compared to 8% Arg-NaF and NaF (p < 0.05). The proportion of *S. sanguis* in the biofilms treated with the Arg-NaF and NaF slurries was similar (p > 0.05) and significantly higher than DIW (p < 0.05). The *S. gordonii* proportional composition was significantly lower for biofilms treated with DIW than all the dentifrice slurries (p < 0.05). Overall, the results of the *3-species* biofilm bacterial composition treated with different test solutions indicated that 2% Arg-NaF maintained an ecological homeostasis by inhibiting *S. mutans* growth and enriching growth of *S. sanguis* and *S. gordonii*.

### Gene expression profile

**Figure 8:**
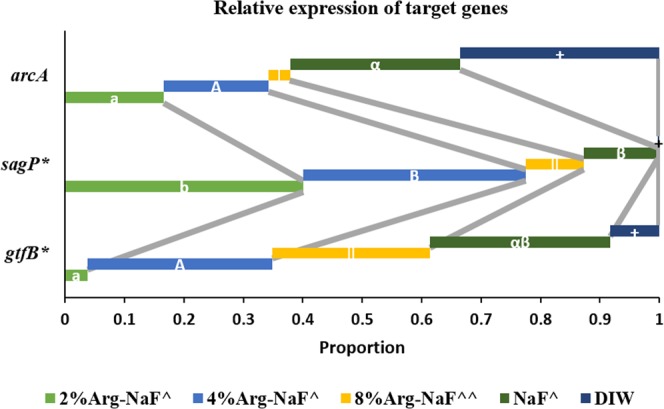
Target gene expression in 3-species biofilm (*S.mutans, S.sanguis, S.gordonii*) treated with different test agents (2%, 4%, 8% Arg-NaF, NaF, and DIW) represented as proportions of relative gene expression for (*gtfB, sagP, arcA)*. The lowercase letters (a,b), uppercase letters (A,B), numerals (I, II), Greek alphabets (α,β), signs (+) represent differences between different gene expressions analyzed by Kruskal-Wallis H test with Dunn’s post-hoc test. The signs i.e. ^,^^, * represent differences between different test groups. Statistical differences for genes: gtfB - 2% Arg-NaF < 4% Arg-NaF; sagP – 2% Arg-NaF, 4% Arg-NaF, NaF > DIW. The factors analysis was done using Friedman’s ANOVA with Dunn’s post-hoc test (Factor 1: Groups; Factor 2: Genes).

Figure 8 presents the relative expression of genes – *gtf*B, *sag*P and *arc*A. Friedman’s non-parametric ANOVA showed a statistically significant difference in the treatment groups (p < 0.001) and genes (p = 0.002). Considering the relative genes expression, the *gtf*B expression for 2% Arg-NaF treated biofilms was significantly lower than 4% Arg-NaF (p = 0.014) but similar to NaF; while *sag*P expression for 2% Arg-NaF, 4% Arg-NaF, NaF was significantly higher than DIW-treated biofilms (p = 0.001). There was no significant difference in *arc*A expression of biofilms treated with different test solutions (p = 0.101).

The forbye analysis further identified that for both 2% Arg-NaF and 4% Arg-NaF-treated biofilms, *sag*P expression was significantly higher when compared to *gtf*B and *arc*A (p < 0.001). The relative gene expression profile of treated biofilms indicated that 2% Arg-NaF downregulated the expression of virulent *gtf*B gene and significantly upregulated the expression of *sag*P with relative dominance of *arc*A, thereby demonstrating a promising anti-caries approach to counter virulent cariogenic biofilms and possible maintenance of ecological homeostasis.

## Discussion

Based on the findings, the null hypothesis in the present study was rejected. It was observed that 2% Arg-NaF exhibited an enhanced antimicrobial effect on cariogenic *S. mutans* biofilms (mono-/3-species) with concurrent enrichment of *S. sanguis* and *S. gordonii –* ADS positive bacteria and an additional biofilm disrupting effect compared to the control – NaF toothpaste (alone). The observation was supported by the microbial cell viability using WST-8 assay, biofilm imaging (SEM and CLSM) and DNA/RNA extraction with subsequent PCR analysis. In this study, a mono-/3-species biofilm model simulating biofilms covering white spot carious lesions in sucrose-exposure conditions was adopted. Being non-mutans streptococci and ADS-positive bacteria, *S. sanguis* and *S. gordonii* are early colonizers that still form major bacterial group in white spot carious lesions and hence, were used in this study. *S. mutans*, a virulent cariogenic bacteria, was included in the study since its proportion in plaque covering early enamel carious lesions is higher as compared to healthy tooth sites^2^.

In the present study, WST-8 assay showed no significant difference in microbial viability among the 2% Arg-NaF, 4% Arg – NaF, 8% Arg – NaF and NaF groups suggesting that increasing the concentrations of arginine in NaF toothpaste had no effect on microbial viability of mono-species biofilm. It is quite possible that the microbial viability will be affected in multi-species biofilm as arginine is known to enrich alkali-producing bacteria by utilizing ADS system with concurrent suppression of *S. mutans* as found in previous studies^3,15,30^.

The biofilm imaging results indicated that both 2% Arg-NaF and 4% Arg-NaF had a biofilm disrupting effect on mono-species *S. mutans* and 3-species biofilm. The disrupting effect in 3-species biofilm is mainly seen in *S. mutans* biofilms. This is beneficial from the remineralization aspect, as presence of biofilm can reduce the effect of anti-caries agent^10^. Additionally, it has been shown in a previous study that 2% Arg-NaF enhanced the remineralization effect of NaF toothpaste^29^. Therefore, it appears that 2% Arg-NaF might have a potential anti-caries effect considering its enhanced antibacterial effect on cariogenic biofilms.

The biofilm disrupting effect that 2% Arg-NaF and 4% Arg-NaF has demonstrated in the present study could be due to either the formation of arginine-fluoride complex, which further enhanced the cytoplasmic acidification of *S. mutans* by fluorides or the ecological pressure exerted by the abundance of *S. sanguis* and *S. gordonii* with the supplementation of arginine. It has been shown that L-arginine supplementation can modify extracellular polymeric substances (EPS) with a reduction in the number of *S. mutans* in mixed-species biofilm due to an increase in the number of *S. gordonii*^31^. The thwarting effect on *S. mutans* outgrowth by L-arginine as explained by the study could be true in this study since a 3-species-biofilm model comprising *S. mutans*, *S. sanguis* and *S. gordini* was adopted. However, the increased ecological pressure encountered could be due to *S. sanguis* as opposed to *S. gordini* because the bacterial dominance of *S. sanguis* was higher compared to *S. gordonii*. Similarly, the gene expression of *sagP* was significantly higher as compared to *arcA* in 2% Arg-NaF-treated multi-species biofilm. Also for 2% Arg-NaF, this explanation is obvious as reflected by the live/dead bacteria ratio computed from CLSM of mono-species biofilm, whereby 2% Arg-NaF demonstrated a significant reduction in *S. mutans* and a predominant enhancement of *S. sanguis* and mild but contributory enrichment of *S. gordonii*.

The total bacterial composition analyzed quantitatively using real-time PCR indicates that 2% Arg-NaF might provide a substantial ecological homeostasis by inhibiting the growth of *S. mutans* with simultaneous increase in the proportion of *S. sanguis* and *S. gordonii*. These results are in concordance with those of a previous study which showed that NaF/Arginine suppressed *S. mutans* and subsequent EPS production^23^. However, on close analysis, it is quite evident that *S. mutans* was still dominant in the composition, irrespective of the treatment, which the biofilms had received. This could be due to the perpetual source of sucrose provided to the biofilms, as the growth of *S. mutans* increased in the presence of sucrose^32^. The model used for the present study simulated conditions in high caries-risk patients and thus justifies the use of 2% Arg-NaF for better ecological homeostasis when compared to the control NaF toothpaste. The enhanced inhibitory effect on *S. mutans* by 2% Arg-NaF and the subsequent enrichment of *S. sanguis* and *S. gordonii*, as explained by the evaluations in mono-species biofilm microbial viability and biofilm imaging, could be the potential reasons.

Moreover, it has been shown that in the presence of arginolytic commensal streptococci, the growth and virulence of cariogenic pathogens are inhibited, which can be an additional explanation of the observed effect of 2% Arg-NaF on the studied multi-species biofilm^12^. It is also noteworthy, that 4% Arg-NaF had similar effect on total bacterial composition in the biofilm as that of 2% Arg-NaF. This shows that 4% Arg- NaF had similar antimicrobial effect as 2% Arg-NaF in a multi-species biofilm. However, in a previous study evaluating the combined remineralization effect of Arg-NaF toothpaste, 4% Arg-NaF did not demonstrate statistically significant remineralization effect when compared to NaF toothpaste^29^. Thus, the combined results of this and the previous study suggest that 2% Arg-NaF has a potential anti-caries effect. The results of the present study further support that 2% Arg-NaF might substantially contribute to ecological homeostasis and biofilm disruption in high-risk patients.

*S. mutans* is identified as cariogenic mainly due to its acidogenic and aciduric properties. During dental caries development, sucrose (fermentable carbohydrates) is used for the production of exopolysaccharides (mostly insoluble glucans) through the bacterial (*S. mutans*) exo-enzyme glucosyltransferases (*gtfs*). *S. mutans* activity with *gtfB* is known to increase during cariogenic shift of the biofilm^33^. The *sag*P (*S. sanguinis)* and *arc*A *(S. gordonii)* are ADS-positive genes involved in the arginine metabolism through ADS activity^19,21^. It has been shown that arginine supplementation can increase the expression of *arc*A gene when compared to no supplementation to biofilms^31^. Moreover, the *arc*A and *sag*P expressions are increased in caries-free subjects when compared to caries-affected subjects^19,21^. Hence, the increased expression of *arc*A and *sagP* genes with subsequent suppression of *gft*B is desirable for effective caries management. In the present study, 2% Arg-NaF significantly inhibited the expression of *gtf*B gene with concurrent significantly enhanced expression of *sag*P gene. The effect on *arc*A expression was statistically similar to that of *gtf*B but was relatively dominant. The gene expression in the 2% Arg-NaF-treated biofilms signified that the treatment had a direct effect on *gtfs*, which might inhibit the production of insoluble glucans^32^, modifying the EPS matrix considerably. This could also be the factor responsible for the observed biofilm disrupting effect of 2% Arg-NaF. Also, the gene expression in the 2% Arg-NaF-treated biofilms provided new insights that supplementation of arginine at 2% by wt. enhanced expression of *sag*P, but did not induce considerable a*rc*A expression as found in a previous study^31^. Therefore, in a multi-species biofilm, the effect of arginine on *S. sanguis* (*sag*P) might be predominant when compared to *S. gordonii* (*arc*A).

Further studies are required to understand how 2% Arg-NaF might behave in a long-term biofilm model, specifically highlighting its effect on biofilm matrix and bacterial interaction. Additionally, clinical exploration or bioreactor simulations with more complex inoculum of the tested formulations in the current study might provide different insights, since the study is *in vitro* and has inherent limitations. Considering the availability of arginine-fluoride combination in a commercial dentifrice, increasing arginine concentrations by weight eventually had reduced F concentrations. The preparation technique was based on our previous study which examined the remineralization potential of the combinations examined in the present study^29^. Despite the limitations, the present study shows how arginine-NaF blend can further enhance the anti-caries effect of NaF toothpaste (alone) with an optimum (2% by wt.) concentration of arginine as opposed to 8% arginine that might further impair the existing effect of NaF. Also, this study confirms that NaF toothpaste alone might not have substantial antimicrobial effect on multi-species biofilm, especially in high caries-risk patients and thus demands supplementation with a biofilm modifier whereby arginine can play a pivotal role.

## Methods

### Preparation and characterization of test agents

A NaF toothpaste (Colgate Triple Action, Colgate-Palmolive Company, USA), milled arginine (L-arginine monohydrochloride, Sigma-Aldrich, St. Louis, USA) and sterilized deionized water (DIW) were used to prepare fresh dentifrice slurry for biofilm treatment as per concentrations listed in Table 1. A standard ratio of 1:3 (dentifrice with/without arginine:DIW) was vortexed at 60 s at room temperature to prepare homogenized aqueous solution. The homogenized solution was then centrifuged at 4000 rpm for 20 mins (Hitachi high-speed centrifuge CR22N, Japan) at 25 °C. The sediment was discarded and the supernatant was transferred to a sterile tube for biofilm treatment and characterization^34^.

**Table 1:** Arginine-Fluoride concentrations of test solutions.

Groups	Fluoride concentration (w/v)	Arginine concentration (w/w)
2% Arg-NaF	0.147%	2%
4% Arg-NaF	0.144%	4%
8% Arg-NaF	0.138%	8%
NaF	0.150%	—
DIW	0.00005%	—

The freshly prepared test arginine-NaF solutions were analyzed for UV-visible characteristics because a notable change in color (as compared to NaF alone) was identified after incorporating the arginine. Randomly selected test solutions during different experimental phases were transferred to 96-well microplate to measure absorbance for UV-visible spectra (200–900 nm) at every 5 nm step increment using microplate reader^35^. The values thus obtained for each group-specific toothpaste supernatant was then summarized as mean to assess spectrum.

### Bacterial strains and mono-species/3-species biofilm model

*S. mutans* UA159 (ATCC 700610), *S. sanguis* DSS-10 (ATCC 10556), and *S. gordonii* DL1 (ATCC 35105) were cultured at 37 °C under anaerobic condition (85% N_2_, 10% H_2_, 5% CO_2_) in brain heart infusion broth (BHI). Cell pellet of *S. mutans*, *S. sanguinis*, *S. gordonii* was adjusted to a concentration of 10^7^ CFU/ml in BHI for biofilm growth.

Artificial saliva-coated hydroxyapaptite (sHA) discs (5 mm in diameter × 2 mm thickness; Clarkson Chromatography Products, Inc., South Williamsport, PA, USA) were used for biofilm growth. The disc dimensions were matched to estimate clinical dimensions of early enamel carious lesions. The manufacturer delivered HA discs were autoclaved and inserted in sterilized (autoclaved) artificial saliva (Phenol red, 4% NaOH, CaCl_2_, MgCl_2_.H_2_O, KH_2_PO_4_, KCl, HEPES, NaN_3_ - 1 ml/disc) for 1 h in incubator at 37 °C to simulate salivary pellicle formation. Mono-species and 3-species biofilm (inoculum ratio - 1:1:1 of *S. mutans: S. sanguis: S. gordonii*) were inoculated on sHA discs with BHI containing 1% sucrose (adjusted to pH 7) in anaerobic chamber (85% N_2_, 10% H_2_, 5% CO_2_) for 24 h. The biofilms were then dip-washed in sterile PBS (1 ml/disc) before receiving treatment with the test solutions. The treatment regimen for group-restricted test solutions (toothpaste supernatant) was: Group 1: 2% arginine – NaF (0.147% F) (2% Arg-NaF), Group 2: 4% arginine – NaF (0.144% F) (4% Arg-NaF), Group 3: 8% arginine – NaF (0.138% F) (8% Arg-NaF), Group 4 (toothpaste supernatant control): NaF (0.15% F) and Group 5: sterilized DIW. The biofilms were then transferred to the respective treatment solutions (180 µl/disc) for 1 min with constant agitation on a shaking incubator (80 rpm, 37 °C). The biofilms were then dip-washed in 1 ml/disc PBS for further transfer to wells containing BHI with 1% sucrose for 24 h anaerobic incubation (85% N_2_, 10% H_2_, 5% CO_2_). After 24 h, the biofilms were subjected to different characterizations. The group-specific mono-species biofilm were subjected to microbial cell viability assay, scanning electron microscopy (SEM) and confocal imaging. The 3-species biofilm were quantified for bacterial composition by DNA extraction and real-time polymerase chain reaction (PCR) analysis, SEM, confocal laser scanning microscopy, and RNA isolation for quantitative reverse-transcription real-time PCR analysis.

### Microbial cell viability in mono-species biofilm

For each test group, inoculated treated biofilms on three sHA discs were transferred to a sterile tube with 1 ml PBS further vortexed for 60 s to receive biofilm suspensions. Aliquots of suspension were used for colorimetric microbial viability detection using a microbial viability assay kit (Dojindo Laboratories, Japan) – water-soluble tetrazolium salt (WST-8). Prior to assay, the coloring reagent was freshly prepared by mixing 9 parts of WST-8 solution with 1 part of diluted electron mediator reagent (mPMS) with dimethyl sulfoxide (DMSO). A 190-µl suspension aliquot was transferred to each well of a 96-well microplate followed by addition of 10-µl coloring reagent. The microplate was incubated for 2 h at 37 °C. After incubation, the absorbance was measured at 450 nm using microplate reader. The assay was performed in duplicate per biofilm suspension with repetitions at three time-points.

### Scanning electron microscopy

Surface topographic biofilm (mono-species/3-species) assessment was done using SEM (SU1510, Hitachi, Japan) at 15 kV, 6000x. The biofilms were fixed with 2.5% glutaraldehyde in PBS (1 ml/disc) overnight. The biofilms were then serially dehydrated with graded series of ethanol concentrations (70%, 85%, 95%, 100%) at every ½ h. The biofilms were then air-dried at room temperature for 1 h, following which were subjected to sputtering (MSP-2S, IXRF systems, USA) with palladium and platinum (120 secs/disc). The sHA discs with inoculated biofilms were mounted on stubs for further analysis. Images subjected to qualitative assessment were captured at different randomly selected areas within the confines of the discs at 6000-x magnification.

### Confocal imaging of biofilms

Confocal laser scanning microscopy (CLSM) of biofilms was done with CLS Biological Microscope (Olympus, FLUOVIEW FV1000, USA) – a two-photon laser scanning microscope. Prior to initializing scan, the treated biofilms were stained with LIVE/DEAD^®^ *BacLight*^™^ Bacterial Viability Kit (SYTO 9/propidium iodide – 1:1 solution in DMSO) L7012 (Invitrogen detection technologies, USA) for 15 mins incubated at room temperature in dark. The biofilms on the sHA discs were transferred to circular coverslips mounted on the fluorescence microscope. Images were taken at 100-x magnification with three randomly selected view fields per biofilm. The images were then examined with Leica QWin v. 2.6. (Leica Microsystems Imaging Solutions, Germany) to determine the percent area with live and dead cells. A ratio of live/dead cells was calculated. The image examination to identify live/dead cells was done in triplicate per biofilm.

### Total bacterial composition in 3-species biofilm

Biofilms were transferred to 1 ml PBS sterile tube to receive 60 s vortexed biofilm suspensions. The biofilm suspension was centrifuged at 7600 g for 10 mins. Then, the supernatant was decanted. The pellet was resuspended in 50 mM EDTA at pH 8.0 with 20 mg/ml lyzoyme and kept in water bath at 37 °C for 1 h. Then, the QIAamp Mini DNA kit (Qiagen, Hilden, Germany) was used to perform the DNA extraction as per manufacturer instructions. DNA extracted from *S. mutans* (ATCC 700610), *S. sanguis* (ATCC 10556) and *S. gordonii* (ATCC 35105) were used as the positive controls. The primers used for real-time PCR in the present study are listed in Table 2. Oligoneucleotide primers (Table 2) and probes (*S. mutans* P is 5′-FAM-TGGAAATGACGGTCGCCGTTATGAA-TAMRA-3′) were used (Applied Biosystems, USA). For each real-time PCR reaction (*S. mutans*), 20 µl of a mixture containing 5 µl of biofilm suspension, 1x Taqman Universal PCR Mix (Applied Biosystems, USA), 200 nM of forward (F) and reverse (R) primers and 250 nM Taqman Probe was prepared. The reaction was performed using Step One Plus (Applied Biosystems, USA). The cycle condition was: 50 °C/2 mins; 95 °C/10 mins; 50 cycles of 95 °C/15 sec and 58 °C/1 min. The standard curve was performed with the DNA of control ATCC *S. mutans* strain containing 5 × 10^3^ – 1 × 10^7^ CFU/ml. For each *S. sanguis* and *S. gordonii* real-time PCR reaction, 20 µl of a mixture containing 5 µl of biofilm suspension, 1x SYBR Green Master Mix (Applied Biosystems, USA) and 200 nM of F/R primers were used. The reaction was performed using Step One Plus (Applied Biosystems, USA). The cycle condition was: 95 °C/20 s; 50 cycles of 95 °C/3 s and 58 °C/30 s; and following melt-curve: 95 °C/15 s; 60 °C/1 min. The standard curve was performed with the DNA of control ATCC *S. sanguis* and *S. gordonii* strain containing 1 × 10^4^ – 1 × 10^9^ CFU/ml.

**Table 2:** Primers used for real time PCR.

Target	Primer *F/R sequence
*S. mutans*	F: 5′-GCCTACAGCTCAGAGATGCTATTCT-3′
	R: 5′-GCCATACACCACTCATGAATTGA-3′
*S. sanguis*	F: 5′-CAAAATTGTTGCAAATCCAAAGG-3′
	R: 5′-GCTATCGCTCCCTGTCTTTGA-3′
*S. gordonii*	F: 5′-CGGATGATGCTAATCAAGTGACC-3′
	R: 5′-GTTAGCTGTTGGATTGGTTGCC-3′
*S. mutans* 16S rRNA	F: 5′-CCTACGGGAGGCAGCAGTAG-3′
	R: 5′-CAACAGAGCTTTACGATCCGAAA-3′
*S. sanguis* 16S rRNA	F: 5′-AAGCAACGCGAAGAACCTTA-3′
	R: 5′-GTCTCGCTAGAGTGCCCAAC-3′
*S. gordonii* 16S rRNA	F: 5′-AAGCAACGCGAAGAACCTTA-3′
	R: 5′-GTCTCGCTAGAGTGCCCAAC-3′
*gtf*B	F: 5′-AGCAATGCAGCCAATCTACAAAT-3′
	R: 5′-ACGAACTTTGCCGTTATTGTCA-3′
*sag*P	F: 5′-GTGGTGGTGGCAATATCGTAG-3′
	R: 3′-CGACCTCGAACCAATTCACTTCC-5′
*arc*A	F: 5′-GTCTTTGACCTCACCAGAAA-3′
	R: 5′-ACTCACGAATAGCCACTTTAG-3′

*F – forward, R – reverse.

### Relative target gene expression

Three sHA discs per test group inoculated biofilms were vortexed for 60 s in 1 ml of PBS to receive suspensions. The suspension was centrifuged at 7600 g for 10 mins. Then, the supernatant was decanted. The pellet was re-suspended in freshly-prepared TE buffer (10 mM Tris-HCl, 1 mM EDTA, pH 7.0) containing 0.4 mg/ml lyzoyme and kept in water bath at room temperature for 15 mins. Then, the SV Total RNA Isolation kit (Promega, Madison, USA) was used to extract the RNA as per manufacturer instructions. The purity of RNA was verified by absorbance measurement at A_260/280_ and run by gel electrophoresis. cDNA was synthesized using Superscript II reverse transcriptase (Invitrogen detection technologies, USA) as per manufacturer instructions. Oligoneucleotide primers (Applied Biosystems, USA) as listed in Table 2 were used to quantify relative gene expression^31^. The relative quantification of *gtf*B, *sag*P, and *arc*A genes was performed using 16S rRNA as a reference gene; an internal control for quantification. For each real-time PCR reaction, 20 µl of a mixture containing 5 µl of suspension, 1x SYBR Green Master Mix (Applied Biosystems, USA) and 200 nM of F/R primers were used. The reaction was performed using Step One Plus (Applied Biosystems, USA). The cycle condition was: 95 °C/20 s; 50 cycles of 95 °C/3 s and 58 °C/30 s; and following melt-curve: 95 °C/15 s; 60 °C/1 min including appropriate negative and positive controls.

### Statistical analysis

All experiments were repeated at least three times at disparate points. The statistical analysis of obtained data collocated in MS Office Excel 2016 (Microsoft Office Professional Plus 2016, Microsoft, USA) was done using SPSS v. 24 (IBM SPSS^®^ Statistics Inc, USA). Data was assessed for normality and homogeneity of variances using Shapiro-Wilk test and Levene’s test, respectively. The statistical differences amongst the test groups for microbial cell viability was determined using Kruskal-Wallis One-way ANOVA with Dunn’s post-hoc test. The data for live/dead bacteria ratio was log transformed for homogeneity of variances and conversion to achieve the vital (live) cells existence subtracted by dead bacteria. The log-transformed data was further analyzed using one-way ANOVA followed by Student-Newman-Keuls (SNK) test for multiple comparisons. DNA and RNA quantified by real-time PCR in 3-species biofilm was subjected to parametric (factors: treatment and bacteria) and non-parametric (factors: treatment and gene) two-way ANOVA with post-hoc tests, respectively. The level of statistical significance for all tests was at 2-tailed p-value < 0.05.

## Conclusion

Within the limitations of the *in vitro* study, the following conclusions are drawn:

1. The incorporation of 2% arginine in NaF toothpaste significantly enhances the antimicrobial effect against caries-generating bacteria (*S. mutans*) when compared to NaF (alone), while 4% and 8% arginine in NaF toothpaste were ineffective in enhancing the antimicrobial effect of NaF.
2. The 2% Arg-NaF toothpaste might maintain better ecological homeostasis by upregulating the non-mutans streptococci (*S. sanguis* and *S. gordonii*).
3. The 2% Arg-NaF toothpaste significantly downregulates the expression of virulent *gtf*B gene and upregulates the expression of *sag*P with relative dominance of *arc*A.
